# Prognostic significance of epidermal growth factor receptor and programmed cell death-ligand 1 co-expression in esophageal squamous cell carcinoma

**DOI:** 10.18632/aging.204535

**Published:** 2023-02-20

**Authors:** Guoxiang Jiang, Yandong Miao, Zhenbo Wang, Qi Zhang, Ping Zhou, Fang Zhang

**Affiliations:** 1 Department of Oncology Radiotherapy, Yantaishan Hospital, Yantai 264025, Shandong, China; 2 Department of Oncology, Yantai Affiliated Hospital of Binzhou Medical University, Yantai 264000, Shandong, China; 3 Department of Radiation Oncology, Binzhou Affiliated Hospital of Binzhou Medical University, Binzhou 256603, Shandong, China; 4 Department of Pathology, The First Hospital of Zibo, Zibo 255200, Shandong, China; 5 Department of Radiation Oncology, Yantai Affiliated Hospital of Binzhou Medical University, Yantai 26400, Shandong, China

**Keywords:** esophageal cancer, squamous cell carcinoma, PD-L1, EGFR, targeted therapy

## Abstract

Our study aimed to observe the correlation between epidermal growth factor receptor (EGFR) and programmed cell death-ligand 1 (PD-L1) expression and evaluate prognostic potential of their co-expression in esophageal squamous cell carcinoma (ESCC) patients. EGFR and PD-L1 expression were evaluated by immunohistochemical analysis. We revealed that there was a positive correlation between EGFR and PD-L1 expression in ESCC (*P* = 0.004). According to the positive relationship between EGFR and PD-L1, all patients were divided into four groups: EGFR (+)/PD-L1 (+), EGFR (+)/PD-L1 (−), EGFR (−)/PD-L1 (+), and EGFR (−)/PD-L1 (−). In 57 ESCC patients without surgery, we found that EGFR and PD-L1 co-expression were statistically correlated with a lower objective response rate (ORR) (*p* = 0.029), overall survival (OS) (*p* = 0.018) and progression-free survival (PFS) (*p* = 0.045) than those with one or none positive protein. Furthermore, PD-L1 expression has a significant positive correlation with infiltration level of 19 immune cells, EGFR expression was significantly correlated with infiltration level of 12 immune cells. The infiltration level of CD8 T cell and B cell were negatively correlated with EGFR expression. On the contrary with EGFR, the infiltration level of CD8 T cell, and B cell were positively correlated with PD-L1 expression. In conclusion, EGFR and PD-L1 co-expression could predict poor ORR and survival in ESCC without surgery, indicating a subset of patients who may benefit from a combination of targeted therapy against EGFR and PD-L1, which may expand the population benefiting from immunotherapy and reduce the occurrence of hyper progressive diseases.

## INTRODUCTION

Esophageal cancer (EC) has the tenth incidence and sixth cancer mortality worldwide, with an estimated 604,100 new cases and 544,076 new deaths per year in the world [1]. At present, the main treatments for EC include surgery, radiotherapy, chemotherapy, and targeted therapy. However, due to limited curative effects and serious adverse reactions, the results are still unsatisfactory [2]. Byiringiro et al. have reported that the overall median survival of 5170 patients who underwent esophagectomy was 42 months [3]. Pai et al. analyzed 126 patients with neoadjuvant chemoradiotherapy and found that 3-year overall survival (OS) is 45%~54% and disease free survival (DFS) 34%~37% [4]. Thus, new prognostic markers and therapeutic strategies are urgently needed.

The epidermal growth factor receptor (EGFR) is a 170-kDa transmembrane receptor and belongs to the ERBB growth factor receptor family. Epidermal growth factor (EGF) is its ligand. After the binding of the two, the receptor will dimerize or heterodimerize, and then autophosphorylate, which activates the downstream pathways, such as PI3K-AKT-mTOR, finally triggering the signal cascade of cell proliferation, differentiation, and survival [5, 6]. It was reported that a high EGFR gene copy number was associated with advanced stage, more lymph node metastasis, and shorter survival time in EC patients [7]. Consistently, EGFR overexpression and amplification were often seen in esophageal squamous cell carcinoma (ESCC) and associated with advanced stage and shorter survival [8].

Interestingly, some studies found that the expression of programmed cell death-ligand 1 (PD-L1) was upregulated by EGFR. Zhang et al. [9] confirmed that in ESCC cell lines with EGFR high expression when the EGFR signal was activated, the expression of PD-L1 was significantly increased, and when the EGFR tyrosine kinase inhibitor was applied, the expression was significantly inhibited. Similarly, Ng et al. [10] demonstrated that the expression of PD-L1 was upregulated by EGFR and its regulation was through the EGFR/ERK pathway in ESCC. By activating the EGFR signal, the expression of PD-L1 increased significantly in an EGFR-dependent manner, and when the EGFR signal was blocked, the expression of PD-L1 dropped sharply. EGFR–AKT, EGFR–Erk, and EGR–PLC-γ signaling pathways may upregulate the expression of PD-L1 [11].

It has been proved that PD-L1 is a ligand of the programmed cell death protein 1 (PD-1) and is expressed in many kinds of tumor cells. PD-L1 on tumor cells binds to the PD-1 receptor on T cells. After the combination, it inhibits the migration and proliferation of T cells and helps tumor cells escape from host immune surveillance, which causes the immune system to be unable to kill tumor cells, thus promoting their growth [12, 13]. Previous research has revealed that PD-L1 was overexpressed and was associated with poor clinical outcomes in ESCC patients [14–16].

Nonetheless, the relationship between EGFR and PD-L1 and the prognostic value of their co-expression are not yet known in ESCC patients. We observed the correlation between EGFR and PD-L1 expression and also assessed the prognostic potential of their co-expression in ESCC patients. Our study aimed to provide a new basis for determining potential prognostic predictors and the combination of targeted EGFR therapy and immunotherapy targeting PD-1/PD-L1, which may expand the population benefiting from immunotherapy and reduce the occurrence of hyper-progressive diseases.

## RESULTS

### Patient clinicopathologic characteristics

A total of 154 ESCC patients (136 men and 18 women) were enrolled in this research retrospectively. The basic condition and clinical characteristics of the patients were summarized in Table 1. The median age of the patients was 65 years (range, 43–92 years) at the date diagnosed. There were 97 patients receiving esophagectomy and 57 patients without esophagectomy.

**Table 1:** Demographic and clinicopathologic features of 154 ESCC patients and correlation with PD-L1 expression.

**Parameters**		**No. of cases (Percentage)**	**PD-L1**		***P* value**
Age (years)	≤65	88 (57%)	55	41	0.133
	>65	66 (43%)	26	32	
Sex	Male	136 (88%)	65	63	0.317
	Female	18 (12%)	16	10	
TNM stage	I–II	59 (38%)	24	36	0.012
	III–IV	95 (62%)	57	37
T stage	T1/2	68 (44%)	28	9	0.001
	T3/4	86 (56%)	53	64
Status of lymph nodes	Negative	59 (38%)	23	36	0.008
	Positive	95 (62%)	58	37	
Primary tumor location	Upper	31 (20%)	10	21	0.030
	Middle	58 (38%)	35	22	
	Lower	65 (42%)	36	30	
EGFR expression	Negative	98 (64%)	43	55	0.004
	Positive	56 (36%)	38	18	
Smoking history	No	53 (34%)	29	24	0.272
	Yes	101 (66%)	52	49	
Alcohol history	No	75 (49%)	39	36	0.885
	Yes	79 (51%)	42	37	
Surgery	No	57 (37%)	20	37	0.001
	Yes	97 (63%)	61	36	

### The expression of EGFR and PD-L1 proteins

**Figure 1:**
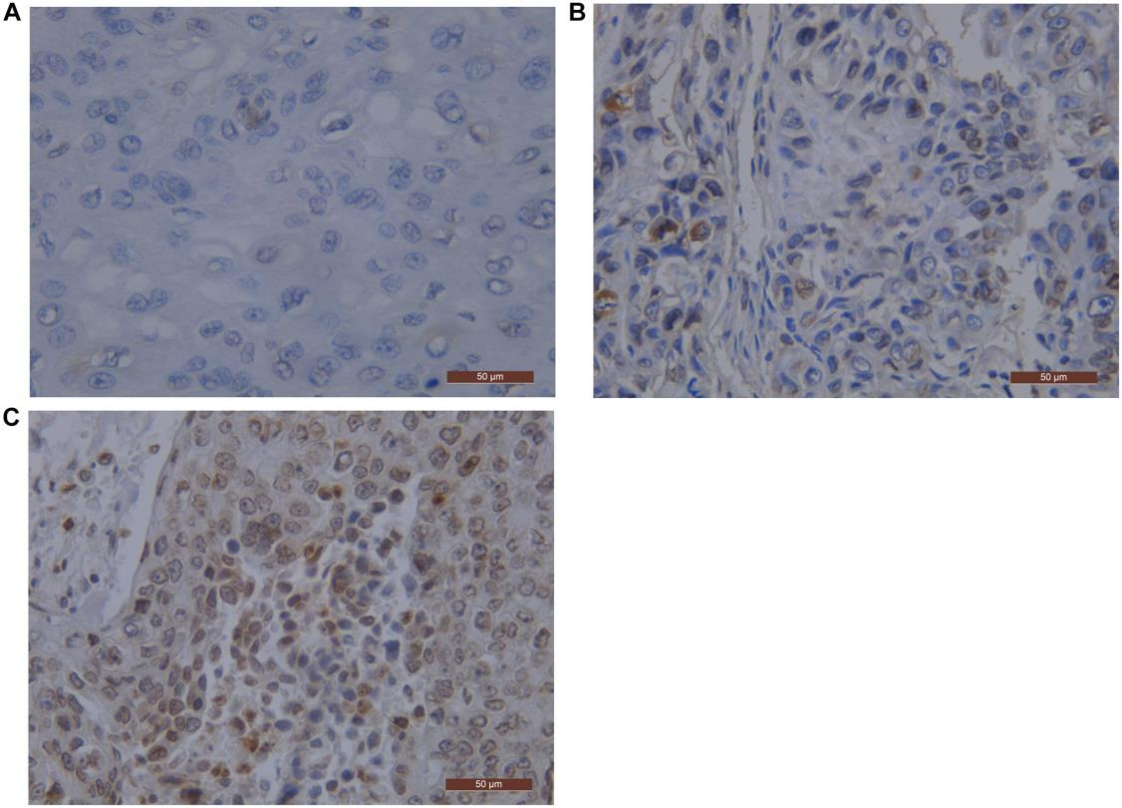
**EGFR representative pictures by immunostaining in ESCC.** Scale bar represents 50 μm. (**A**) Staining score 0 negative pattern for EGFR; (**B**) Staining score 2 negative pattern for EGFR; (**C**) Staining score 12 negative pattern for EGFR. Abbreviations: EGFR: Epidermal Growth Factor Receptor; ESCC: Esophageal Squamous Cell Carcinoma.

**Figure 2:**
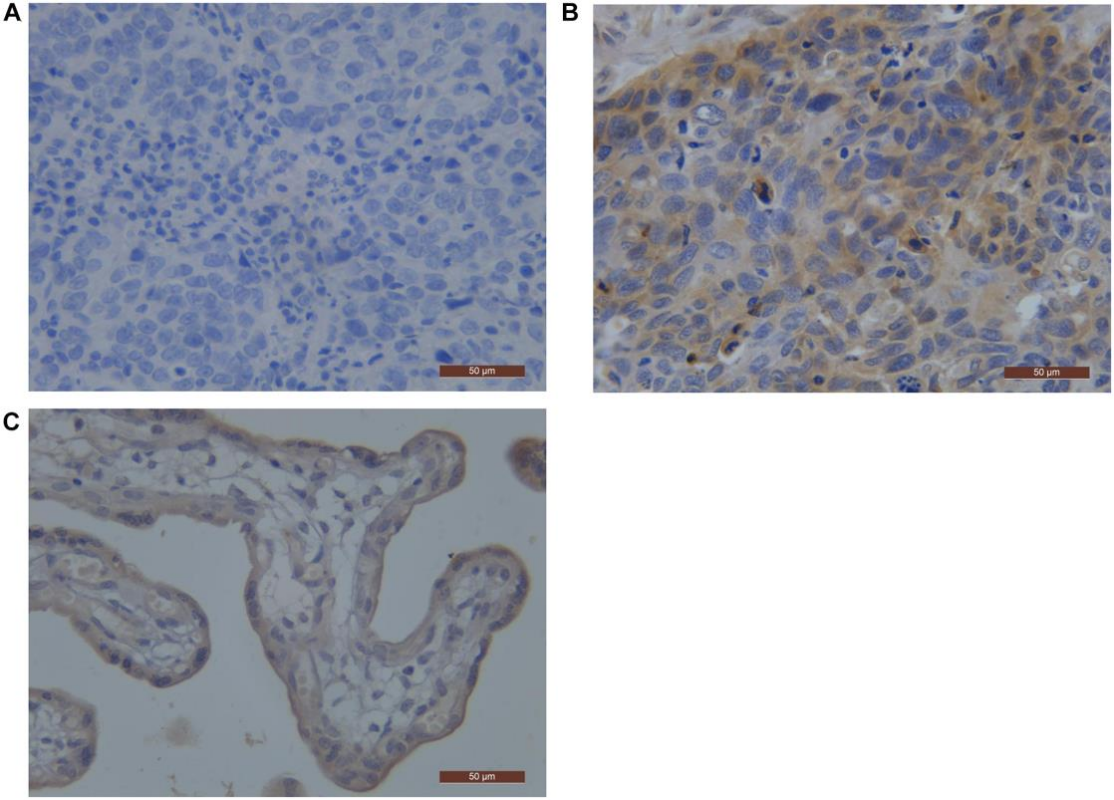
**PD-L1 representative pictures by immunostaining in ESCC.** (**A**) Negative pattern for PD-L1; (**B**) Positive pattern for PD-L1; (**C**) the positive control staining (Placental tissue). Abbreviations: ESCC: Esophageal Squamous Cell Carcinoma; PD-L1: Programmed Death-Ligand 1.

The positive rates of EGFR and PD-L1 were 36.4% (56/154) and 52.6% (81/154), respectively by IHC staining. Among all ESCC patients, 38 (24.7%) patients were EGFR (+)/PD-L1 (+), 18 (11.7%) patients were EGFR (+)/PD-L1 (−), 43 (27.9%) patients were EGFR (−)/PD-L1 (+), and 55 (35.7%) patients were EGFR (−)/PD-L1 (−). Representative PD-L1 and EGFR staining patterns are shown in Figures 1 and 2. We revealed that there was a positive correlation between EGFR and PD-L1 expression (*P* = 0.004) (Table 1).

### Differential expression analysis of EGFR and PD-L1 in ESCC

The analysis by Sento academic online tool showed that the expression of the EGFR gene was higher in unpaired and paired ESCC tissues than in corresponding normal tissues, but there is no statistical difference (*P* > 0.05, Supplementary Figure 1A, 1B). The expression of the PD-L1 gene was significantly higher in unpaired ESCC tissues than in corresponding normal tissues, but there is no statistical difference in the paired group (Supplementary Figure 1C, 1D).

### The relationship between EGFR and PD-L1 with clinicopathologic characteristics

In our study, we analyzed the relationship between EGFR and clinical characteristics, and the correlation between PD-L1 and patients’ characteristics, respectively. Table 1 showed that surgery, TNM stage, T stage, the status of lymph nodes, and primary tumor location were related to PD-L1 expression (*P* < 0.05). Table 2 showed that the T stage, the status of lymph nodes, and primary tumor location were related to PD-L1 expression (*P* < 0.05). Apart from them, no obvious correlation was observed between EGFR and PD-L1 expression with clinicopathologic characteristics, as shown in Tables 1 and 2.

**Table 2:** The correlation between epidermal growth factor receptor with clinicopathologic characteristics in ESCC patients.

**Parameters**		**EGFR**		***P* value**
Age (years)	≤65	31	65	0.177
	>65	25	33	
Sex	Male	47	81	0.839
	Female	9	17	
Tumor location	Upper	6	25	0.020
	Middle	19	39	
	Low	31	34	
TNM stage	I–II	19	41	0.333
	III–IV	37	57	
T stage	T1/2	25	12	0.000
	T3/4	31	86	
Status of lymph nodes	Negative	15	44	0.026
	Positive	41	54	
Smoking history	Yes	37	64	0.923
	No	19	34	
Alcohol history	Yes	26	53	0.361
	No	30	45	
PD-L1	+	38	43	0.004
	−	18	55	
Surgery	No	21	36	0.925
	Yes	35	62	

**Figure 3:**
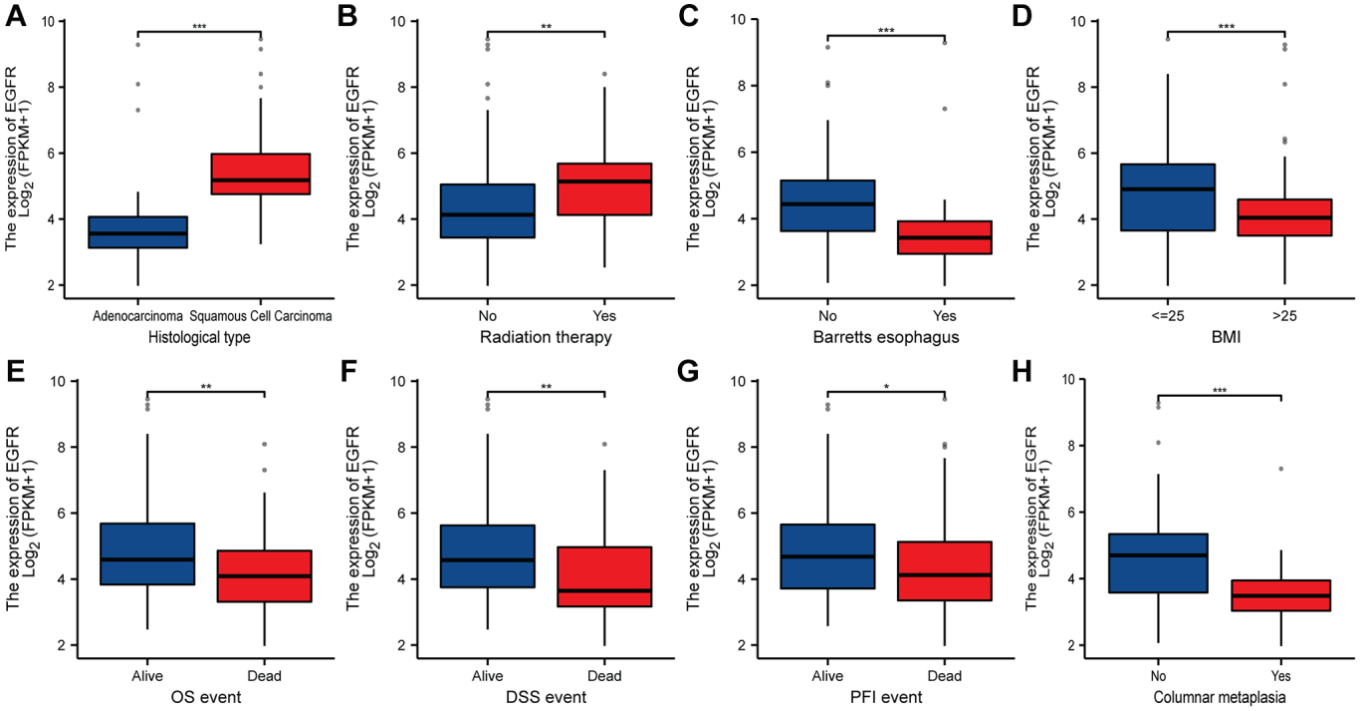
**The relationship between EGFR and clinicopathologic characteristics in ESCC.** (**A**) Histological type. (**B**) Radiation therapy. (**C**) Barretts esophagus. (**D**) BMI. (**E**) OS event. (**F**) DSS event. (**G**) PFI event. (**H**) columnar metaplasia. ^*^, ^**^, ^***^ represents *P* < 0.05, *P* < 0.01, *P* < 0.001, respectively. Abbreviations: BMI: Body Mass Index; OS: Overall Survival; DSS: Disease-Specific Survival; PFI: Progression-Free Interval.

**Figure 4:**
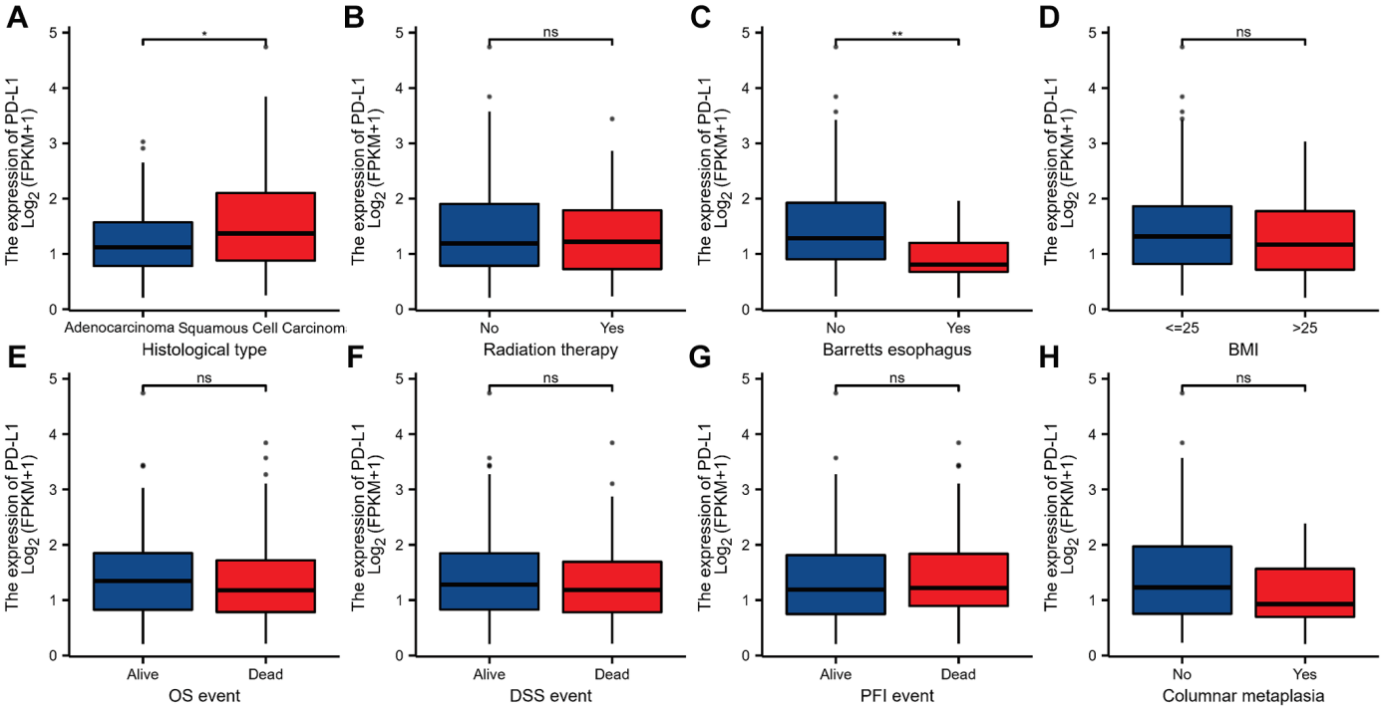
**The relationship between PD-L1 and clinicopathologic characteristics in ESCC.** (**A**) Histological type. (**B**) Radiation therapy. (**C**) Barretts esophagus. (**D**) BMI. (**E**) OS event. (**F**) DSS event. (**G**) PFI event. (**H**) columnar metaplasia. ^*^, ^**^, ^***^ represents *P* < 0.05, *P* < 0.01, *P* < 0.001, respectively. ns represents “No significant”. Abbreviations: BMI: Body Mass Index; OS: Overall Survival; DSS: Disease-Specific Survival; PFI: Progression-Free Interval.

Furthermore, using the public database, we found that EGFR was significantly overexpressed in the following subgroups: squamous cell carcinoma, previously received radiation therapy, non-Barretts esophagus, BMI ≤25, alive patients, and non-columnar metaplasia (*P* < 0.05, Figure 3). Similarly, we found that PD-L1 was significantly overexpressed in the squamous cell carcinoma and non-Barretts esophagus subgroup. But there is no statistical difference in the radiation therapy, BMI, survival event, and columnar metaplasia (*P* > 0.05, Figure 4).

### Survival analysis

**Figure 5:**
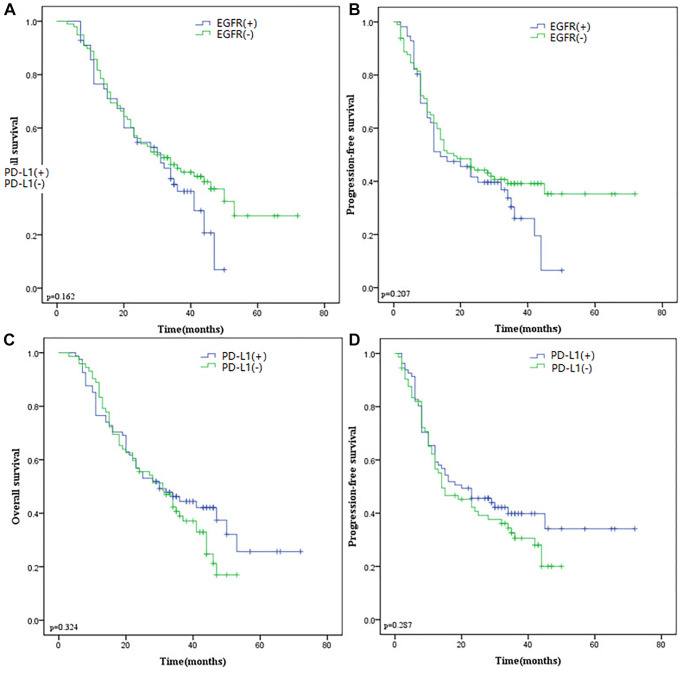
(**A**) Kaplan-Meier curves of OS according to EGFR expression. (**B**) Kaplan-Meier curves of PFS according to EGFR expression. (**C**) Kaplan-Meier curves of OS according to PD-L1 expression. (**D**) Kaplan-Meier curves of PFS according to PD-L1 expression. Abbreviations: OS: Overall Survival; EGFR: Epidermal Growth Factor Receptor; PD-L1: Programmed Death-Ligand 1; PFS: Progression-Free Survival.

**Figure 6:**
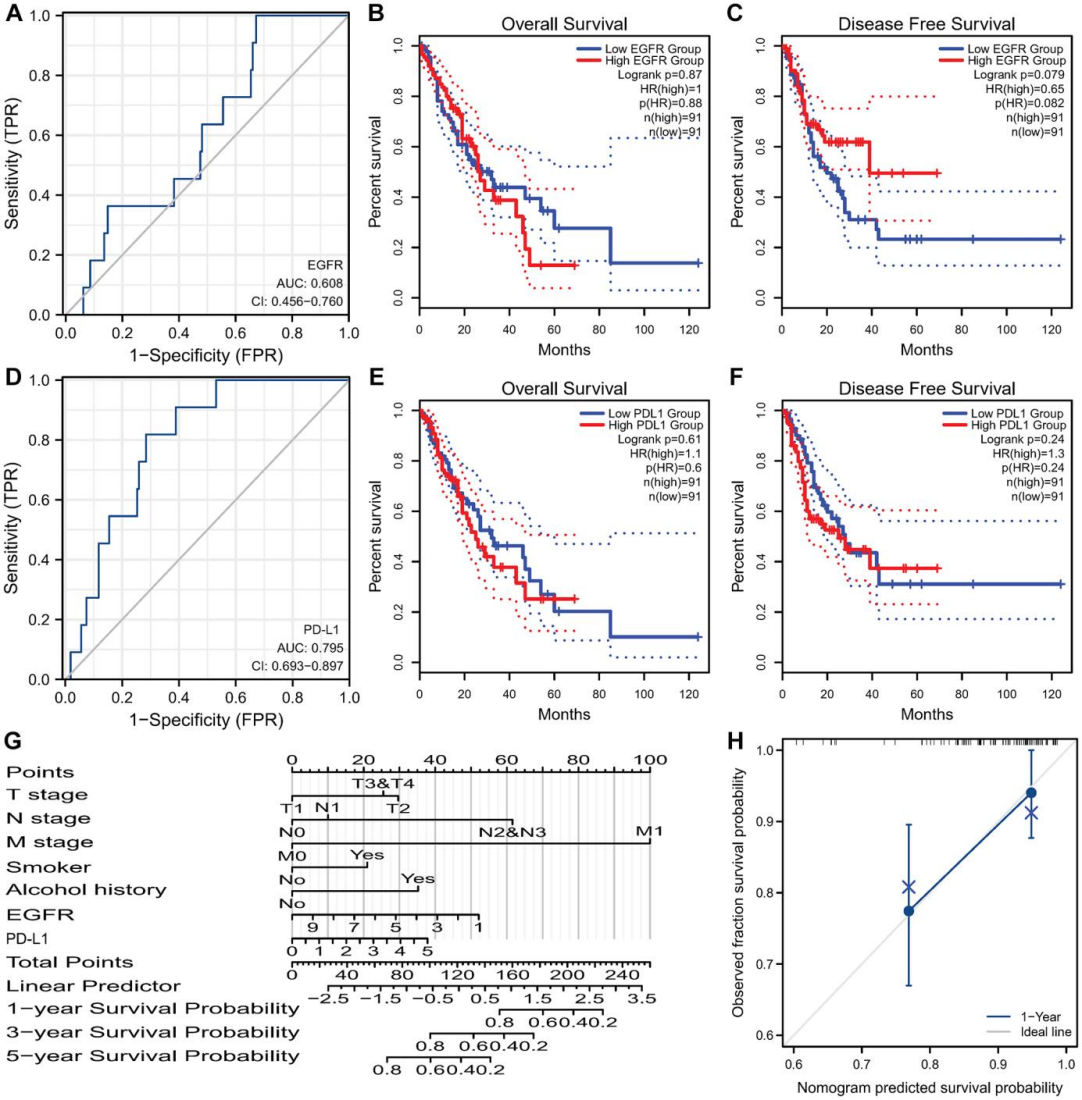
**Survival analysis of EGFR and PD-L1 in ESCC and the nomogram.** (**A**) ROC curves of EGFR gene predicting prognosis. EGFR expression isn’t associated with OS (**B**) and DFS (**C**) in TCGA-ESCC. (**D**) ROC curves of PD-L1 gene predicting prognosis. PD-L1 expression isn’t associated with OS (**E**) and DFS (**F**) in TCGA-ESCC. (**G**) Nomogram for predicting 1-, 3-, and 5-year OS of ESCC based on clinicopathological features and the expression of EGFR and PD-L1. (**H**) Calibration curves of prediction models for 1-year survival of nomograms. Abbreviations: ROC: receiver operating characteristic; EGFR: Epidermal Growth Factor Receptor; OS: Overall Survival; DFS: Disease Free Survival; TCGA: The Cancer Genome Atlas; ESCC: Esophageal Squamous Cell Carcinoma; PD-L1: Programmed Death-Ligand 1.

In the current study, we analyze the relationship between PD-L1 or EGFR with OS and PFS of all ESCC patients. Disappointingly, neither EGFR nor PD-L1 expression was an independent prognostic factor for survival by the Kaplan-Meier method and log-rank test. The result is shown in Figure 5. Similar results are obtained from the survival analysis in the public database (Figure 6A–6F).

The nomogram was used to predict the probabilities of 1-, 3- and 5-year OS by incorporating the TNM stage, smoker history, alcohol history, EGFR expression, and PD-L1 expression. Each factor was assigned a score in proportion to its contribution to the risk of survival. The calibration curve showed that the actual survival time is in agreement with the predicted survival time (Figure 6G, 6H).

### Survival analysis of EGFR and PD-L1 co-expression

**Figure 7:**
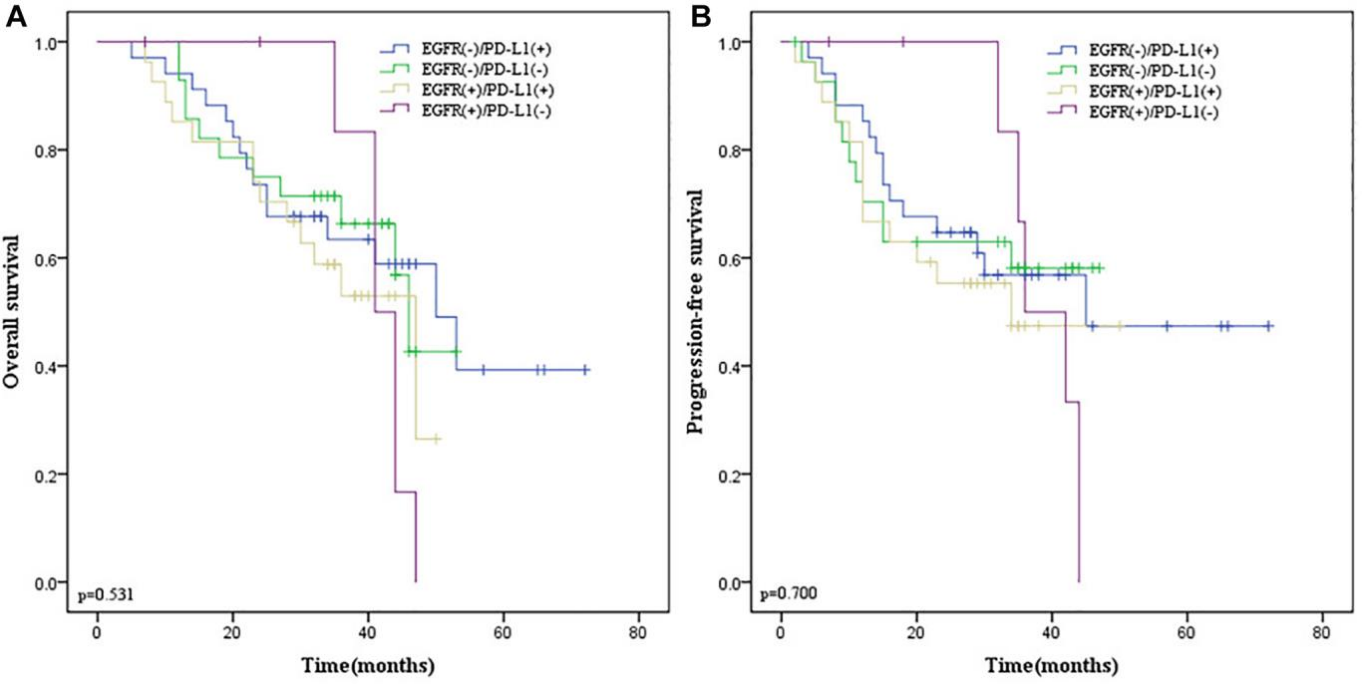
**Kaplan-Meier survival curves according to EGFR and PD-L1 co-expression in ESCC patients.** (**A**) OS survival curves of different subgroups. (**B**) PFS survival curves of different subgroups. Blue line indicates EGFR (−)/PD-L1(+); green line indicates EGFR (−)/PD-L1(−); yellow line indicates EGFR (+)/PD-L1(+); purple line indicates EGFR (+)/PD-L1(−). EGFR, Epidermal Growth Factor Receptor. Abbreviation: PD-L1: programmed death-ligand 1.

According to the positive relationship between EGFR and PD-L1, all patients were divided into 4 groups: EGFR (+)/PD-L1 (+), EGFR (+)/PD-L1 (−), EGFR (−)/PD-L1 (+) and EGFR (−)/PD-L1 (−). We found that EGFR and PD-L1 co-expression were not statistically correlated with OS (*p* = 0.304; Figure 7A) and PFS (*p* = 0.351; Figure 7B) by the Kaplan-Meier method and log-rank test.

### Stratification analysis of EGFR and PD-L1 co-expression

**Figure 8:**
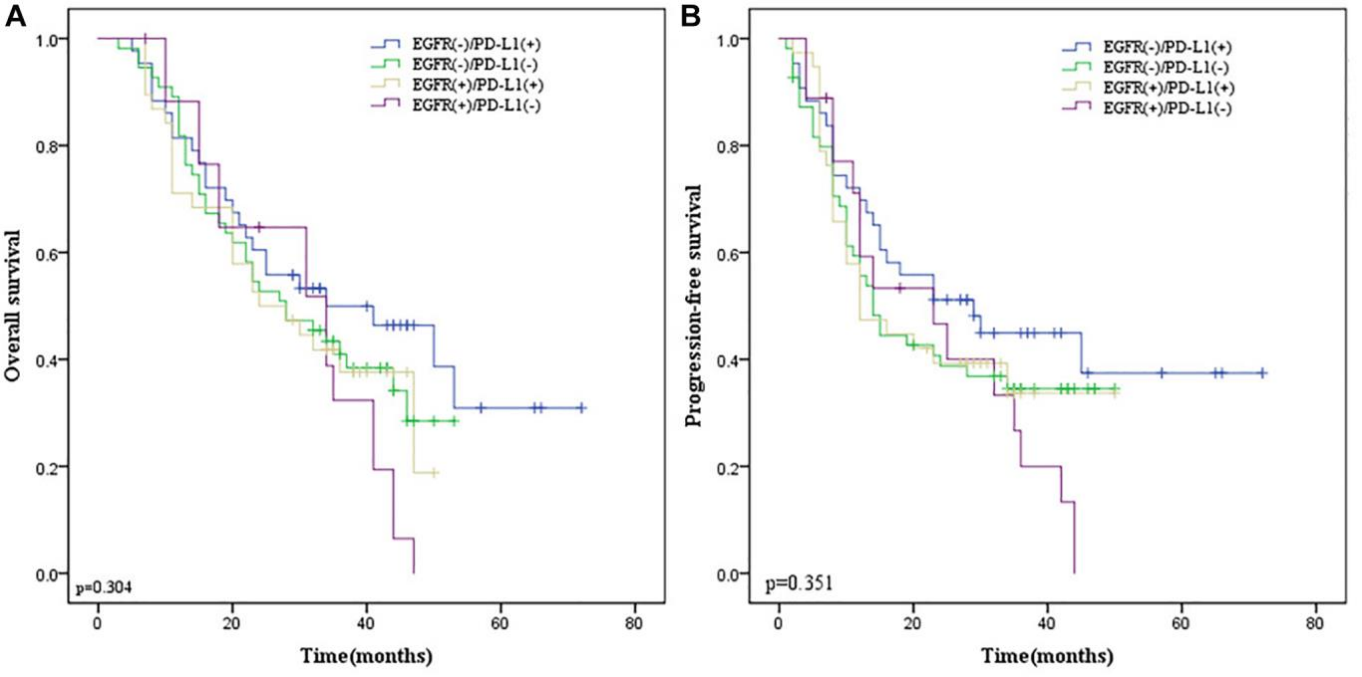
**The Kaplan-Meier survival curves in ESCC patients receiving esophagectomy.** (**A**) OS survival curves according to EGFR and PD-L1 co-expression. (**B**) PFS survival curves according to EGFR and PD-L1 co-expression. Blue line indicates EGFR (−)/PD-L1(+); green line indicates EGFR (−)/PD-L1(−); yellow line indicates EGFR (+)/PD-L1(+); purple line indicates EGFR (+)/PD-L1(−).

**Figure 9:**
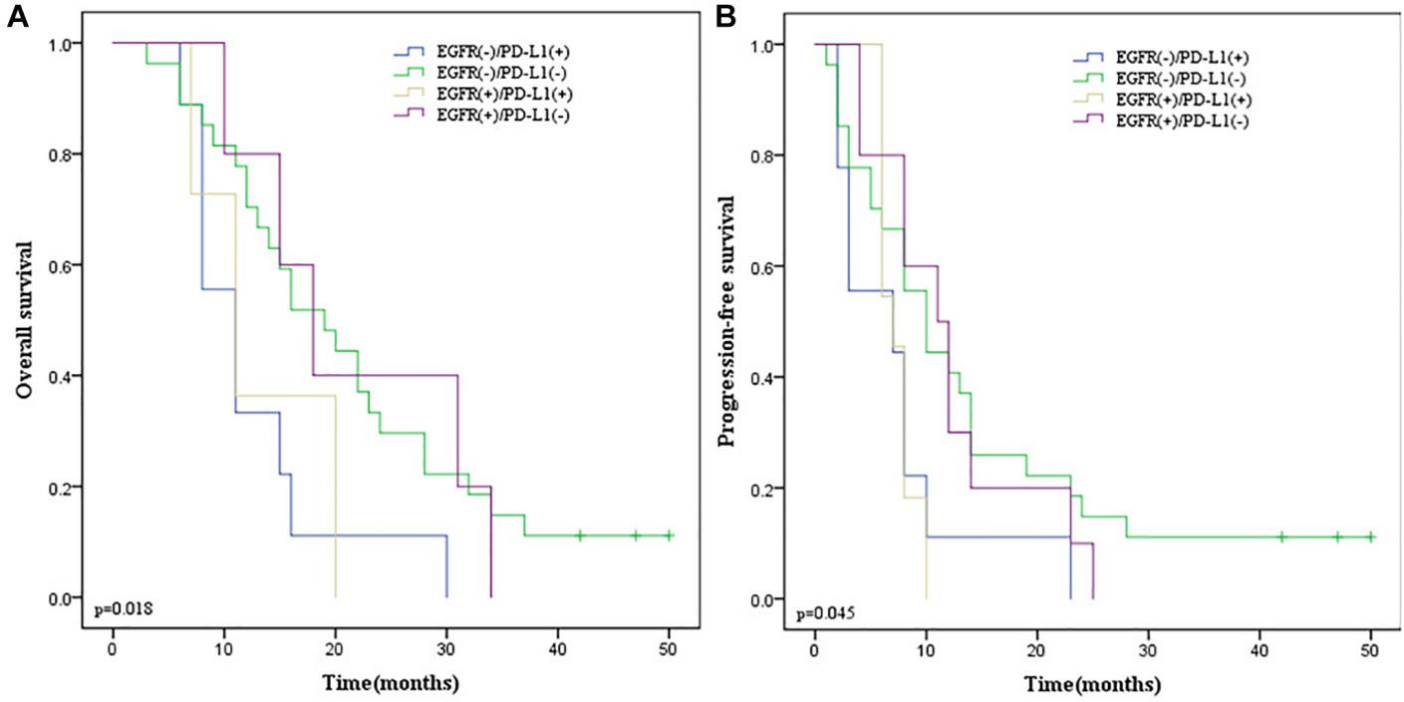
**The Kaplan-Meier survival curves in ESCC patients without esophagectomy.** (**A**) OS survival curves according to EGFR and PD-L1 co-expression. (**B**) PFS survival curves according to EGFR and PD-L1 co-expression. Blue line indicates EGFR (−)/PD-L1(+); green line indicates EGFR (−)/PD-L1(−); yellow line indicates EGFR (+)/PD-L1(+); purple line indicates EGFR (+)/PD-L1(−); Abbreviations: ESCC: Esophageal Squamous Cell Carcinoma; OS: overall survival; EGFR: Epidermal Growth Factor Receptor; PD-L1: programmed death-ligand 1; PFS: Progression Free Survival.

Given that co-expression of EGFR and PD-L1 were not correlated with survival endpoints in all patients and surgery was related to PD-L1 expression, we performed stratification analysis according to whether surgery was performed. In 97 ESCC patients receiving esophagectomy, there was no obvious correlation between their co-expression with OS (*p* = 0.531; Figure 8A) and PFS (*p* = 0.700; Figure 8B). However, in 57 ESCC patients without surgery, we found that EGFR and PD-L1 co-expression were statistically correlated with a lower ORR (*p* = 0.029) by the Chi-square test, inferior OS (*p* = 0.018; Figure 9A) and PFS (*p* = 0.045; Figure 9B) by the Kaplan-Meier method and log-rank test than those with one or none positive protein.

### Correlation analysis of EGFR and PD-L1 immune cell infiltration in ESCC

**Figure 10:**
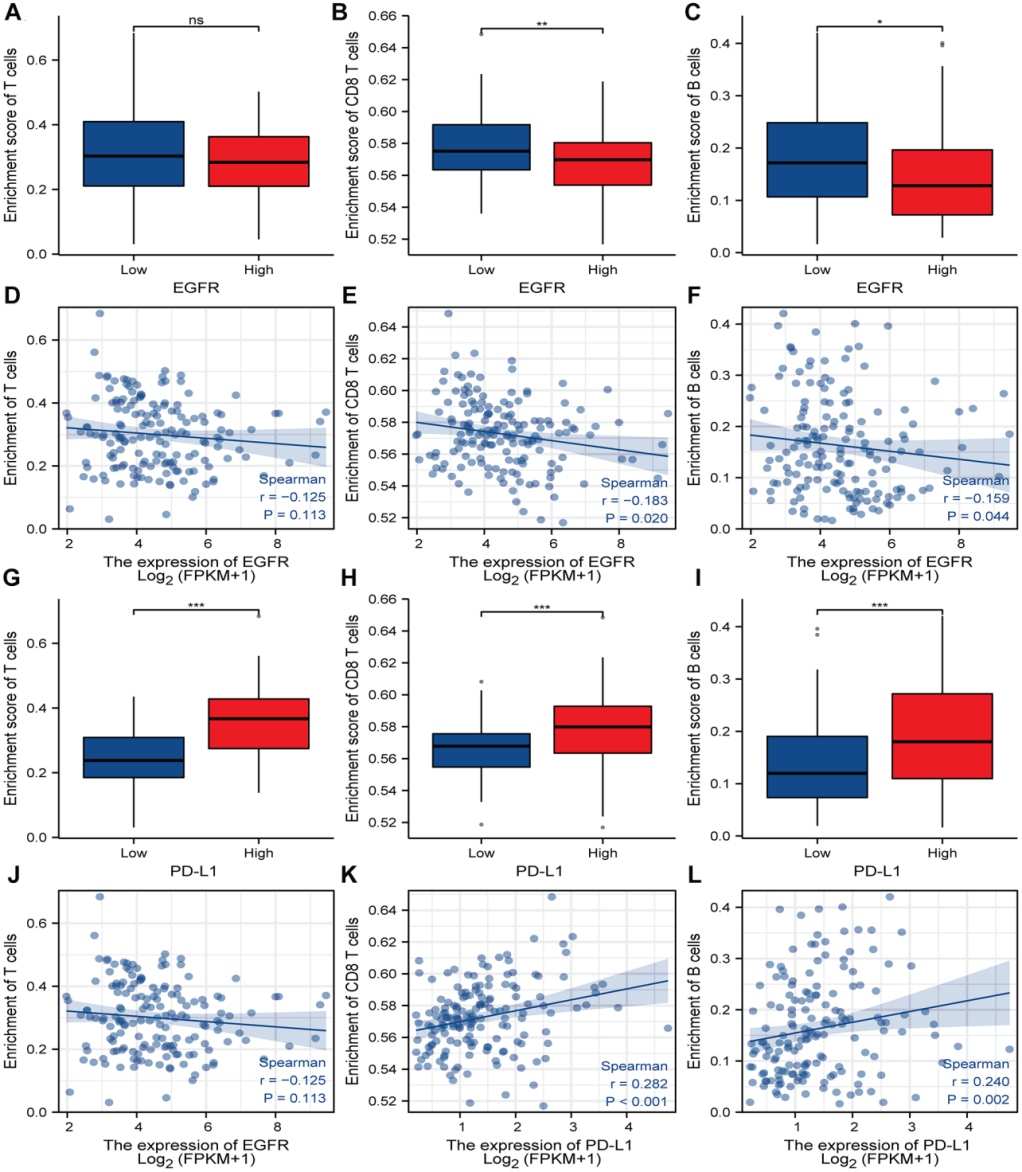
**Correlation analysis of EGFR, PD-L1 expression and immune cell infiltration in ESCC.** (**A**–**C**) Boxplot showing the correlation between enrichment score and the level of infiltration of T cells, CD8 T cells and B cells in high and low EGFR expression subgroups. (**D**–**F**) The immune cell infiltration levels of T cells, CD8 T cells and B cells have correlation with EGFR expression. (**G**–**I**) Boxplot indicate the correlation between enrichment score and the level of infiltration of T cells, CD8 T cells and B cells in high and low PD-L1 expression subgroups. (**J**–**L**) The immune cell infiltration levels of T cells, CD8 T cells and B cells have correlation with PD-L1 expression. A positive value of the correlation coefficient (r) indicates a positive relationship between the two variables, and a negative value indicates a negative relationship. ^*^, ^**^, ^***^ represents *P* < 0.05, *P* < 0.01, *P* < 0.001, respectively. ns represents “No significant”, Abbreviations: EGFR: Epidermal Growth Factor Receptor; PD-L1: programmed death-ligand 1; ESCC: Esophageal Squamous Cell Carcinoma.

As we all know, EGFR is involved in some immune-related pathways and PD-L1 is an immune checkpoint, its expression was correlated with the infiltration level of immune cells. Therefore, to further explore the relationship between EGFR, PD-L1 gene expression, and the infiltration level of immune cells in ESCC, we performed correlation analysis, and the results showed that EGFR gene expression was significantly correlated with the infiltration level of 12 immune cells (Supplementary Figure 2A), of which four cells were positively correlated (Tcm cells, NK CD56dim cells, NK cells, and Tgd cells, Supplementary Figure 2B–2E) and eight cells were negatively correlated (B cell, Mast cells, CD8 T cells, pDC, Tem, NK CD56bright cells, Eosinophils, Th17 cells, Supplementary Figure 2F–2M). The detailed analysis results are shown in Table 3. Interestingly, we found that the enrichment score of T cell, CD8 T cell, and B cell in the EGFR high expression group was lower than that in EGFR low expression group. Besides, the infiltration level of T cell, CD8 T cell, and B cell was negatively correlated with EGFR gene expression (Figure 10A–10F).

**Table 3:** Correlation analysis between expression of EGFR gene and immune cell infiltration in ESCC.

**Gene**	**Immune cells**	**Correlation coefficient** **(Spearman)**	***P* value** **(Spearman)**	**Correlation coefficient** **(Pearson)**	***P* value** **(Pearson)**
EGFR	Th17 cells	–0.492	<0.001	–0.405	<0.001
EGFR	Eosinophils	–0.449	<0.001	–0.384	<0.001
EGFR	Tcm	0.405	<0.001	0.255	0.001
EGFR	NK CD56bright cells	–0.342	<0.001	–0.329	<0.001
EGFR	Tem	–0.323	<0.001	–0.299	<0.001
EGFR	NK CD56dim cells	0.314	<0.001	0.259	<0.001
EGFR	pDC	–0.304	<0.001	–0.245	0.002
EGFR	NK cells	0.234	0.003	0.255	0.001
EGFR	CD8 T cells	–0.183	0.020	–0.189	0.016
EGFR	Tgd	0.165	0.036	0.181	0.021
EGFR	Mast cells	–0.161	0.041	–0.155	0.049
EGFR	B cells	–0.159	0.044	–0.117	0.140
EGFR	Th2 cells	0.150	0.057	0.180	0.022
EGFR	Neutrophils	–0.131	0.096	–0.140	0.075
EGFR	T cells	–0.125	0.113	–0.106	0.178
EGFR	iDC	0.086	0.278	0.063	0.427
EGFR	Macrophages	0.066	0.401	0.080	0.309
EGFR	Th1 cells	0.052	0.512	0.040	0.610
EGFR	aDC	0.050	0.524	0.040	0.613
EGFR	T helper cells	–0.039	0.625	0.042	0.596
EGFR	TFH	–0.035	0.657	–0.018	0.825
EGFR	Cytotoxic cells	0.033	0.677	0.024	0.762
EGFR	TReg	–0.008	0.922	–0.028	0.728
EGFR	DC	0.004	0.964	0.032	0.689

Abbreviations: aDC: Dendritic cells; iDC: immature Dendritic cells; DC: Dendritic cells; NK: Natural Killer cells; pDC: Plasmacytoid DC; Tcm: T central memory; Tem: T effector memory; Tfh: T follicular helper; Tgd: T gamma delta.

PD-L1 gene expression has a significant positive correlation with the infiltration level of 19 immune cells (Supplementary Figure 3A), including Th1, NK CD56dim cells, aDC, cytotoxic cells, T cells, Treg cells, Macrophages, DC, TFH, iDC, Neutrophils, Mast cells (Supplementary Figure 3B–3T). The detailed analysis results are shown in Table 4. On the contrary with EGFR, the enrichment score of T cell, CD8 T cell, and B cell in the PD-L1 high expression group was higher than that in the PD-L1 low expression group. Besides, the infiltration level of T cell, CD8 T cell, and B cell were positively correlated with PD-L1 gene expression (Figure 10G–10L).

**Table 4:** Correlation analysis between expression of PD-L1 gene and immune cell infiltration in ESCC.

**Gene**	**Immune cells**	**Correlation coefficient** **(Spearman)**		***P* value** **(Spearman)**	**Correlation coefficient** **(Pearson)**	***P* value** **(Pearson)**
PD-L1	aDC	0.511		<0.001	0.469	<0.001
PD-L1	B cells	0.24		0.002	0.176	0.025
PD-L1	CD8 T cells	0.282		<0.001	0.258	<0.001
PD-L1	Cytotoxic cells	0.49		<0.001	0.47	<0.001
PD-L1	DC	0.385		<0.001	0.351	<0.001
PD-L1	Eosinophils	–0.026		0.739	–0.051	0.519
PD-L1	iDC	0.346		<0.001	0.321	<0.001
PD-L1	Macrophages	0.407		<0.001	0.357	<0.001
PD-L1	Mast cells	0.304		<0.001	0.233	0.003
PD-L1	Neutrophils	0.341		<0.001	0.276	<0.001
PD-L1	NK CD56bright cells	–0.079		0.316	–0.099	0.211
PD-L1	NK CD56dim cells	0.518		<0.001	0.496	<0.001
PD-L1	NK cells	0.181		0.022	0.138	0.08
PD-L1	pDC	0.172		0.028	0.116	0.14
PD-L1	T cells	0.475		<0.001	0.418	<0.001
PD-L1	T helper cells	0.223	0.004	0.163	0.038	
PD-L1	Tcm	0.27	<0.001	0.25	0.001	
PD-L1	Tem	0.169	0.032	0.069	0.383	
PD-L1	TFH	0.368	<0.001	0.299	<0.001	
PD-L1	Tgd	0.059	0.454	0.071	0.371	
PD-L1	Th1 cells	0.573	<0.001	0.524	<0.001	
PD-L1	Th17 cells	–0.139	0.079	–0.156	0.048	
PD-L1	Th2 cells	0.101	0.2	0.1	0.204	
PD-L1	TReg	0.469	<0.001	0.408	<0.001	

Abbreviations: aDC: Dendritic cells; iDC: immature Dendritic cells; DC: Dendritic cells; NK: Natural Killer cells; pDC: Plasmacytoid DC; Tcm: T central memory; Tem: T effector memory; Tfh: T follicular helper; Tgd: T gamma delta.

## DISCUSSION

Once EGFR and downstream signaling networks are activated, multiple cellular processes such as proliferation, invasion, and metastasis of cells are triggered [17]. EGFR in cancer cells can be activated by two different mechanisms. One is activated by the binding of EGF, TGF-α or amphiregulin to EGFR, and the other is activated by the mutation of tyrosine kinase in EGFR [17]. It was reported that EGFR mutation was rare in EC [7, 18, 19], but EGFR overexpression and amplification were frequently observed in EC [7, 8, 20, 21], indicating activation of EGFR in EC was induced by the binding of EGF, TGF-α, or amphiregulin to EGFR. Therefore, in the current study, we only evaluated EGFR expression in cancer cells by immunohistochemical analysis in ESCC patients and did not detect its mutation. EGFR proteins were positively expressed in 56 (36.4%) patients.

Previous research has confirmed that EGFR activation can induce the expression of PD-L1 by EGFR–PI3K–AKT, EGFR–Erk, and EGR–PLC-γ signal pathways in ESCC cell lines [9–11]. A recent study showed that overexpression of EGFR can mediate the immune escape of tumor cells by upregulating PD-L1 expression in head and neck cancers [22]. Given this relationship, we analyzed the relationship between the expression of EGFR and PD-L1 in ESCC patients. Among all ESCC patients, there was an obvious positive association between PD-L1 and EGFR expression (*P* = 0.004). 38 (24.7%) patients were EGFR (+)/PD-L1 (+), 18 (11.7%) patients were EGFR (+)/PD-L1 (−), 43 (27.9%) patients were EGFR (−)/PD-L1 (+), and 55 (35.7%) patients were EGFR (−)/PD-L1 (−). Activated EGFR signaling can also recruit or reprogram suppressive immunocytes, inhibit major histocompatibility complex (MHC) molecule levels, and upregulate inhibitory cytokines and metabolites, which induces the immunosuppressive tumor microenvironment (TME) [23]. As we all know, CD8+T cell is the most important group of tumor effector cells in specific immune response, and the degree of CD8+T invasion in tumor tissue is positively correlated with immune efficacy. EGFR signal not only prevents the recruitment of effector CD8+T cells but also promotes the infiltration of Treg cells (immunosuppressive cells that help tumors escape), which has a negative effect on the efficacy of ICIs [24, 25]. We also found that the infiltration level of CD8 T cells was negatively correlated with EGFR gene expression. Nishii et al. found that EGFR-mutant NSCLC has a noninflamed TME, with low infiltration by CD8+ T cells [26]. Lu et al. found that the decreases in the density and function of CD8+ TILs were associated with LUAD with EGFR-activating mutations [27]. Chen et al. [28] found that activated EGFR induced Immunoglobulin-like transcript 4 expression and created an immunosuppressive and tumor-promoting TME in non-small cell lung cancer cells. These results indicate that EGFR inhibits anti-tumor immune response by influencing TME. It was a novel mechanism for EGFR-induced immunosuppression. Kato et al. [29] found there was an association between EGFR mutations and time treatment failure durations <2 months. Hyperprogressive disease has been found in 20% of patients with EGFR alterations and has a worse prognosis [30]. Gainor et al. [31] observed that EGFR mutations are associated with low response rates to ICI treatment in lung cancer patients. PD-1 receptor and its ligand PD-L1 are the most important immune checkpoint proteins and are involved in the immune escape of cancer cells [32]. Currently, immunotherapy has quickly become a new treatment option and has altered the paradigm of EC treatment. Moreover, the expression of immune biomarkers can reflect the effect of immunotherapy [33]. In tumor immunity, CD8+T cells are the crucial tumor suppressor cells, which form physical contact with malignant tumor cells and induce tumor cell death by activating intracellular signals [34, 35]. It is well known that the PD-1/PD-L1 axis is a key pathway leading to T-cell exhaustion and the expression of PD-1 on CD8+ T cells is related to a severely exhausted T-cell response [36]. We also found the infiltration level of CD8 T cell and B cell were positively correlated with PD-L1 gene expression. Jansen et al. speculate that the decrease in the lethality of T cells to tumors is due to the excessive number of depleted T cells with positive checkpoints or the high expression of PD-L1 in tumors, but that stem cell-like CD8+T cells form too few anti-tumor bases [37]. Lu et al. reported that the PD-1/PD-L pathway also contributes to T cell and B cell development and activation [38]. Guo et al. found that B cells in the TME were associated with clinical benefits in patients with advanced ESCC receiving anti-PD-1/PD-L1-based therapy [39]. However, a series of clinical trials have shown that the effective rate of immunotherapy is only about 12% to 30% in EC patients [40–43]. Consistent with non-small cell lung cancer patients, the efficacy of immunotherapy in patients with negative driver genes is less than 20% [44, 45], while the response rate in patients with positive driver genes is even lower, and most of them are ineffective [23]. Biomarkers with high predictive value in EGFR wild-type tumors, such as PD-L1 expression, are not fully applicable to EGFR-mutant tumors; and they believed that reasonable combination therapy was necessary [46]. Therefore, considering that EGFR in EC patients is frequently activated [7, 8, 20, 21] and is positively correlated with PD-L1 expression, our study may provide new insight into the combination of targeting EGFR therapy or chemoradiotherapy with PD-1/PD-L1 targeted immunotherapy, which may expand the population benefiting from immunotherapy and reduce the occurrence of hyper progressive diseases.

Previous research reported that EGFR/PD-L1 pairs could distinguish survival between EGFR low/PD-L1 (+) and EGFR high/PD-L1 (−) groups, the median OS time of patients with high EGFR/PD-L1 (−) tumors was much shorter than that of patients with low EGFR/PD-L1 (+) [9]. In our current study, according to the positive relationship between EGFR and PD-L1, all patients were divided into 4 subgroups: EGFR (+)/PD-L1 (+), EGFR (+)/PD-L1 (−), EGFR (−)/PD-L1 (+), and EGFR (−)/PD-L1 (−). Nevertheless, we failed to find a significant association between EGFR and PD-L1 co-expression and prognosis. Our previous findings revealed that PD-L1 was an independent predictor of inferior OS and PFS [47]. Given the positive association between PD-L1 and EGFR expression, we performed stratification analysis according to whether surgery was performed. In 97 ESCC patients receiving esophagectomy, there were no statistical correlations between the co-expression of EGFR and PD-L1 with OS and PFS. However, in 57 ESCC patients without surgery, we found that EGFR and PD-L1 co-expression were statistically correlated with a lower ORR (*p* = 0.029), OS (*p* = 0.018), and PFS (*p* = 0.045) than EGFR (−)/PD-L1 (−), EGFR (+)/PD-L1 (−) and EGFR (−)/PD-L1 (+) subgroups. These inconsistent results suggested that the clinical significance of biomarkers may vary with different treatment regimens.

The reasons for negative data may be as follows. Firstly, this was a retrospective study with relatively small patient samples. Secondly, because all ESCC patients with available cancer specimens were enrolled in our research, random selection was not performed, which may cause additional bias. The third was the different sources of specimens: for patients undergoing esophagectomy, we evaluated resection specimens, while for patients without surgery, we evaluated gastroscopic biopsy specimens. A prospective large sample study should be investigated in the future.

In conclusion, our results show that there was a positive correlation between EGFR and PD-L1 expression in ESCC patients, and for patients without surgery, EGFR and PD-L1 co-expression could predict poor ORR and inferior survival, indicating a subset of patients who may benefit from a combination of targeted therapy against EGFR and PD-L1, which may expand the population benefiting from immunotherapy and reduce the occurrence of hyper progressive diseases.

## MATERIALS AND METHODS

### Bioinformatics analysis

#### Differential expression analysis of EGFR and PD-L1 in ESCC

The Level 3 HTSeq-FPKM format RNAseq data were obtained from The Cancer Genome Atlas (TCGA, https://portal.gdc.cancer.gov/) ESCC Project, then screened for differentially expressed EGFR and PD-L1 in ESCC by the Wilcox test using the R package “limma” [48]. The detailed calculation process and code can be found in Supplementary Documents 1 and 2.

#### Clinical correlation and survival analysis of EGFR and PD-L1 in ESCC

Kruskal-Wallis test was used to perform the clinical correlation analysis between the gene expression (EGFR and PD-L1) and different clinicopathological features Body Mass Index (BMI), columnar metaplasia, histological type, Barretts esophagus, radiation therapy, OS, disease-specific survival (DSS), and progression-free interval (PFI). DSS is defined as the length of time between the initial diagnosis until the date of death due to the diagnosed type of cancer. PFI is the period from the date of diagnosis until the date of the first occurrence of a novel tumor event, which includes a new primary tumor, local recurrence, the progression of the disease, distant metastasis, or death due to the tumor [49]. Survival analysis of EGFR and PD-L1 in ESCC was performed mainly through a database based on the TCGA-ESCC cohort such as GEPIA2 and Kaplan-Meier plotter database. Then, we selected Sento academic online tool (https://www.xiantao.love/) to plot receiver operating characteristic (ROC) curves for EGFR and PD-L1 predicting CRC prognosis, prognostic column line plots as well as calibration analysis.

TNM stage, smoker history, alcohol history, EGFR, and PD-L1expression were used to draw a nomogram by the R-packages “Hmisc,” “lattice,” “Formula,” “ggplot2,” “foreign” and “rms” [50–53]. Calibration traces were used to assess the consistency between the actual and predicted survival rates.

#### Analysis of EGFR and PD-L1 immune cell infiltration in ESCC

The single sample gene set enrichment analysis (ssGSEA) algorithm, built into the R package “GSVA”, (GSVA: gene set variation analysis for microarray and RNA-Seq data) was utilized to evaluate the degree of immune cell infiltration in ESCC and explored the correlation between EGFR and PD-L1 gene expression and the level of immune cell infiltration by Spearman correlation analysis. Lollipop plots were used to demonstrate the relationship between EGFR and PDL1 gene expression and all immune cell infiltration, and correlation analysis between EGFR and PDL1 gene expression and individual immune cell infiltration levels was performed using the Spearman method (*P* < 0.05 indicates statistical significance). The detailed calculation process and code can be found in Supplementary Documents 3–5. Gene Set Enrichment Analysis (GSEA) is considering experimenting with genome-wide expression profiles from two categories of samples, labeled 1 or 2. Sequencing genes based on the correlation between gene expression and category differentiation by using any appropriate measure [54]. ssGSEA was an extension of GSEA, which allows one to define an enrichment score that represents the degree of absolute enrichment of a gene set in each sample within a given data set. The detailed calculation process and code can be found in Supplementary Document 6 [55]. The main types of immune cells are activated Dendritic cells (aDC); immature Dendritic cells (iDC); Dendritic cells; CD8 T cell; B cell; Eosinophils; Macrophages; Mast cell; Cytotoxic cells; Neutrophils; NK CD56bright cells; NK CD56dim cells; Natural Killer cells; Plasmacytoid DC (pDC); T cell; Helper T cells; T central memory (Tcm); T effector memory (Tem); T follicular helper (Tfh); T gamma delta (Tgd); Th1 cells; Th17 cells; Th2 cells; Treg cells [56].

### Patients

We retrospectively collected all EC patients hospitalized in the First Hospital of Zibo and Affiliated Hospital of Binzhou Medical University from January 1, 2015 to December 31, 2018. The inclusion criteria were no history of other malignancies; pathologic confirmation of ESCC; with complete clinicopathological and follow-up information. The exclusion criteria were as follows: non-squamous cell carcinoma and second primary cancer. Consequently, 81 patients were recruited for the present study. According to the seventh edition of the AJCC TNM staging system, the selected patients were staged.

All patients were followed up until death or 31 December 2020. OS: the time from the date of diagnosis to death due to any cause or the last follow-up. Progression-free Survival (PFS) was defined as the time from the start time of treatment to death from any cause or date of the first relapse. Objective Response Rate (ORR) was defined as the percentage of patients whose tumors shrank within a certain period of time, including complete and partial responses. All patients will be followed up regularly, once every 3–6 months in the first 5 years, and once a year thereafter, and follow up at any time if they feel unwell. Routine follow-up examinations include imaging examinations, hematology examinations, and physical examinations.

### Immunohistochemistry

The tissues of ESCC patients were formalin fixed and paraffin embedded. We cut these tissues to a thickness of 3μm for immunohistochemistry (IHC) staining. Briefly, the sections are dewaxed in xylene and rehydrated in descending grades of ethanol, and then blocked. After blocking, we incubate the sections with rabbit anti-PD-L1 monoclonal antibody (1:60, AB205921, Abcam) and rabbit anti-EGFR monoclonal antibody (1:60, Ab52894, Abcam) at 4°C overnight. On the second day, add the second antibody to the slices and incubate at room temperature for 60 min, then observe through DAB system staining. Two experienced pathologists who did not know patients’ conditions independently give reports on the staining results.

### Evaluation of PD-L1 immunostaining

As described previously, the results were estimated as relative percentage staining on tumor cells [9]. According to the instructions, positive control was used to ensure quality control during the IHC evaluation. The proportion of PD-L1 positive cells was estimated as the percentage of total tumor cells: 0, 0%–1%; 1, 1%–5%; 2, 5%–10%; 3, >10% [9, 57, 58]. Five visual fields were selected for the PD-L1 expression score. Calculate the average percentage of PD-L1 positive cells in the five fields of each sample. Expression of PD-L1 in tumor tissues was considered positive if tumor cytoplasmic and membrane staining >5%. If PD-L1 staining of the tumor cell is >5%, it is considered that the expression of PD-L1 is positive [9, 57, 58].

### Evaluation of EGFR immunostaining

The expression of EGFR in tumor tissues was analyzed according to the multiplication of the intensity and the rate of positive cells. If the EGFR score was less than 8 points, it was defined as a low expression, and if the score was greater than or equal to 8 points, it is defined as a high expression. Consistent with previous research, the intensity was classified into 4 types: 0 for negative staining, 1 for weak staining, 2 for moderate staining, and 3 for strong staining, respectively. Record the percentage of positive cells: 0%–25% is recorded as 1; 26%–50% is recorded as 2; 51%–75% is recorded as 3, 75%–100% is recorded as 4 [59].

### Statistical analysis

Chi-square test or Fisher’s exact test was applied for categorical data. The Kaplan–Meier curves and log-rank analysis were used for the analysis and comparison of 3-year OS and PFS. A two-sided test was performed and *P* < 0.05 was considered statistically significant. The statistical software package SPSS version 20.0 (SPSS Inc., Chicago, IL, USA) was used to analyze data.

### Data availability statement

Some or all data during the study are available from the corresponding author by request. (Immunohistochemistry data, OS and PFS).

## Supplementary Materials

Supplementary Figures

Supplementary Document 1

Supplementary Document 2

Supplementary Document 3

Supplementary Document 4

Supplementary Document 5

Supplementary Document 6
