# Supplementary material for: Prognostic significance of epidermal growth factor receptor and programmed cell death-ligand 1 co-expression in esophageal squamous cell carcinoma
Source: Aging (Albany NY). 2023 Feb 20;15(4):1107–29. doi: 10.18632/aging.204535 (PMC10008495; doi:10.18632/aging.204535)
Supplement: Supplementary Figures [file aging-15-204535-s001.pdf]

SUPPLEMENTARY FIGURES

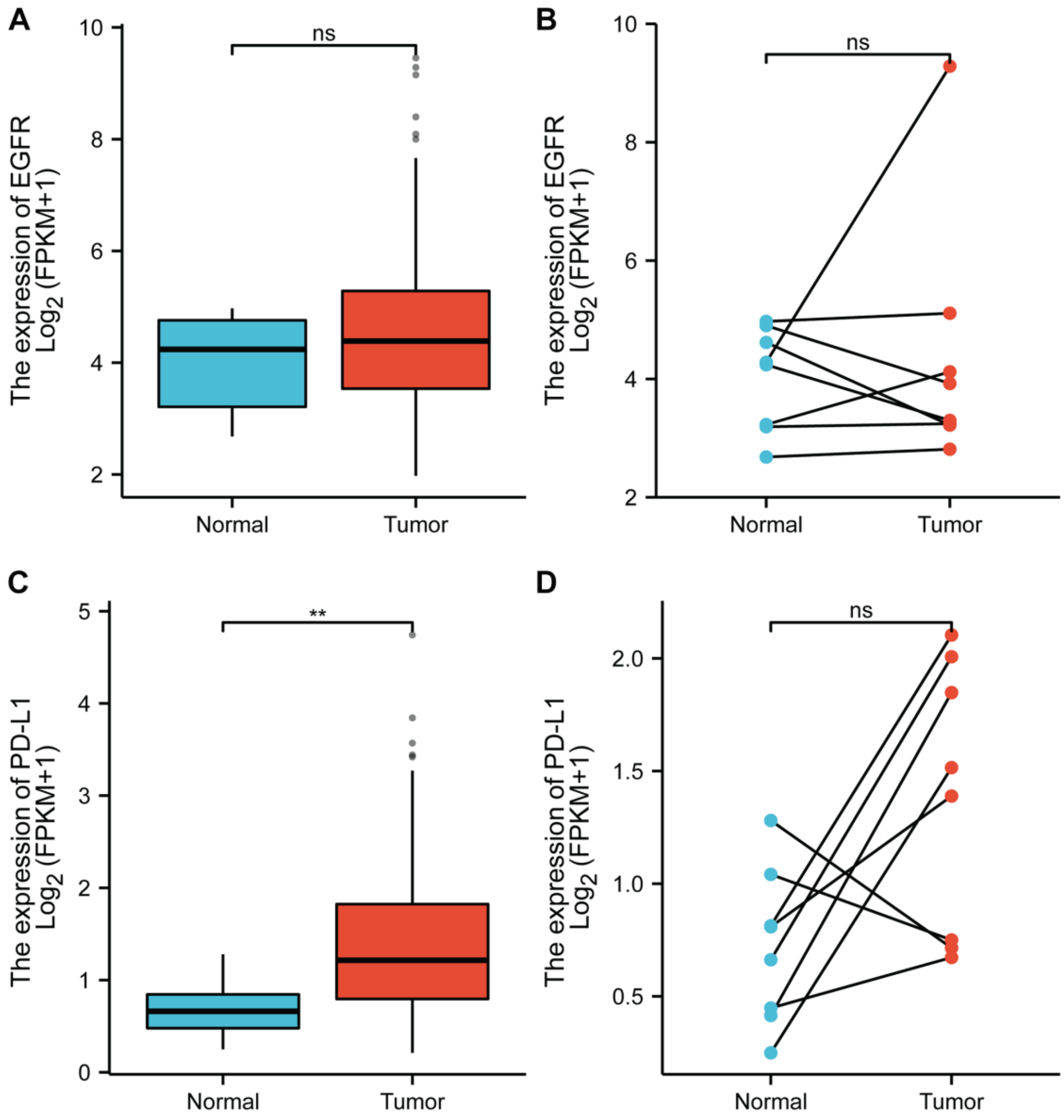

Supplementary Figure 1. Differential expression analysis of EGFR and PD-L1 in normal and ESCC tissues. Differential expression of EGFR in unpaired (**A**) and paired ESCC samples and corresponding normal samples (**B**). Differential expression of PD-L1 in unpaired (**C**) and paired ESCC samples and corresponding normal samples (**D**). *** represents $P < 0.001$.

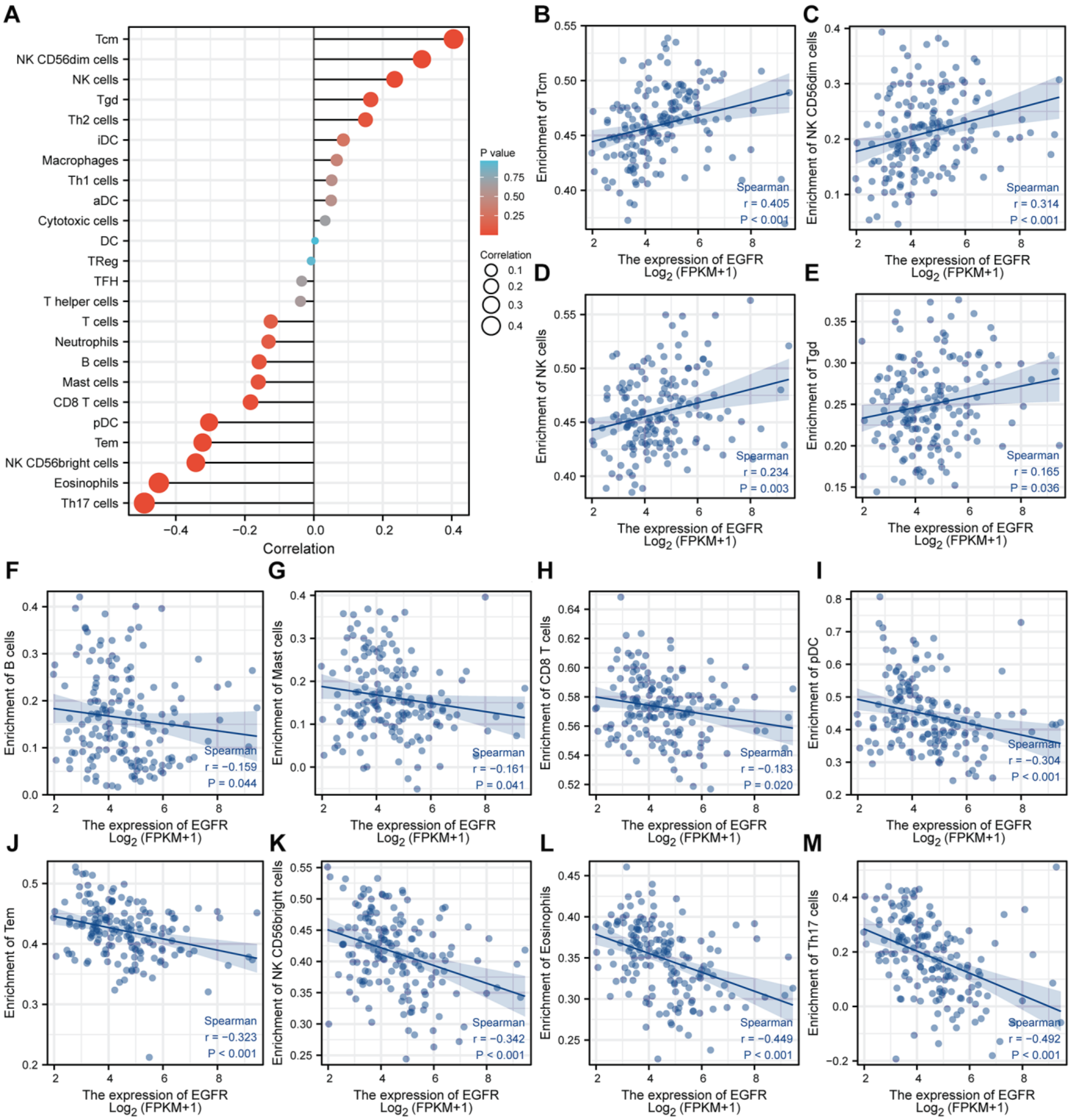

Supplementary Figure 2. Correlation analysis of EGFR expression and immune cell infiltration in ESCC. (**A**) Lollipop plot showing the correlation between EGFR expression and the level of infiltration of 24 immune cells. (**B**–**M**) 12 immune cell infiltration levels have a significant correlation with EGFR expression. (**B**) Tcm, (**C**) NK CD56dim cells, (**D**) NK cells, (**E**) Tgd, (**F**) B cells, (**G**) Mast cells, (**H**) CD8 T cells, (**I**) pDC, (**J**) Tem, (**K**) NK CD56bright cells, (**L**), Eosinophils, (**M**) Th17 cells. A positive value of the correlation coefficient (r) indicates a positive relationship between the two variables, and on the contrary, a negative value indicates a negative relationship. Abbreviations: EGFR: Epidermal Growth Factor Receptor; ESCC: Esophageal Squamous Cell Carcinoma; Tcm: T central memory; NK: Natural Killer cells; Tgd: T gamma delta; pDC: Plasmacytoid DC; Tem: T effector memory; Th17: T helper 17.

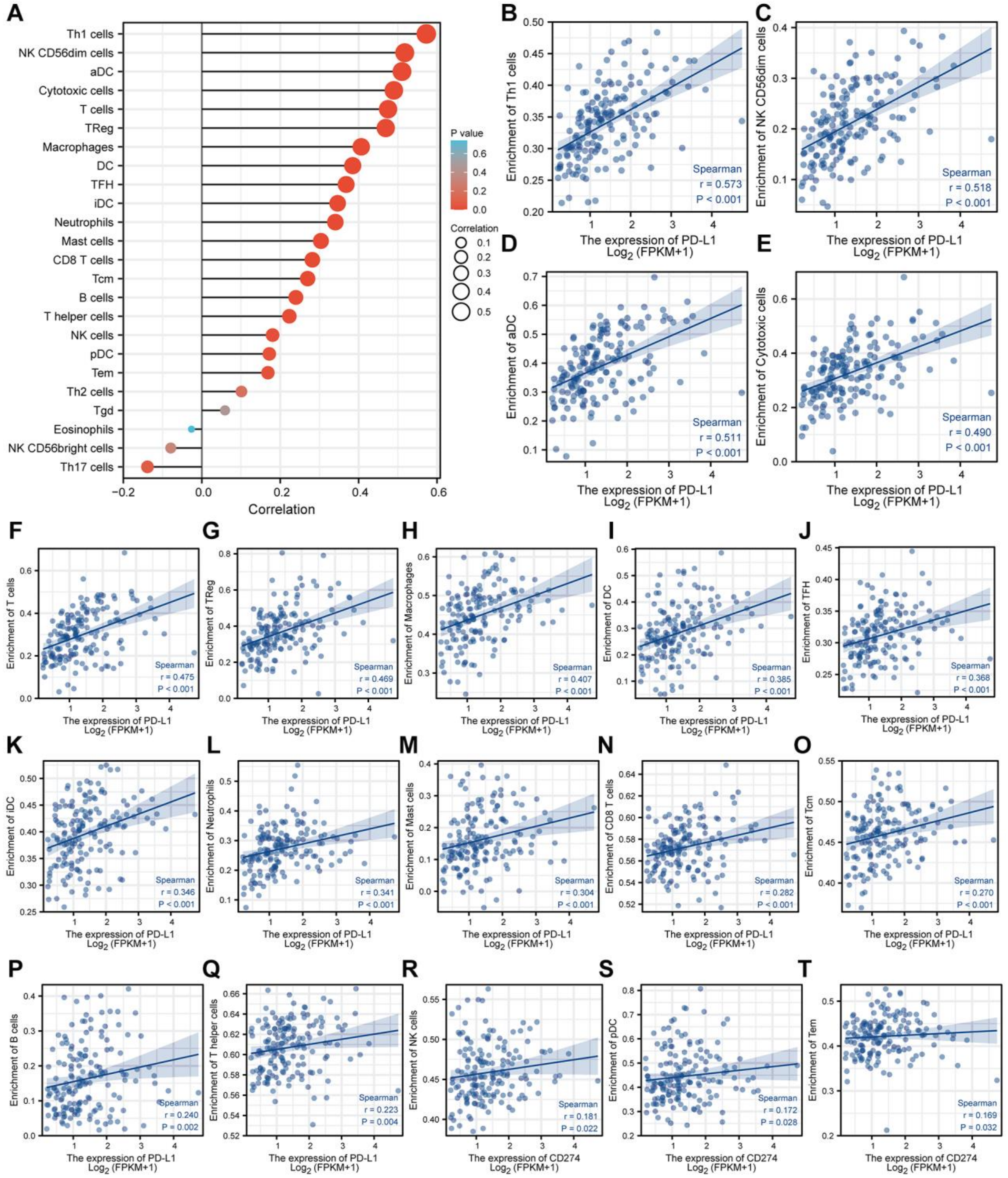

Supplementary Figure 3. Correlation analysis of PD-L1 expression and immune cell infiltration in ESCC. (**A**) Lollipop plot showing the correlation between PD-L1 expression and the level of infiltration of 24 immune cells. (**B**–**T**) 19 immune cell infiltration levels have a significant positive correlation with PD-L1 expression. (**B**) Th1 cells, (**C**) NK CD56dim cells, (**D**) Cytotoxic cells, (**E**) aDC, (**F**) T cells, (**G**) Treg, (**H**) Macrophages, (**I**) DC, (**J**) iDC, (**K**) TFH, (**L**), neutrophils, (**M**) Mast cells, (**N**) CD8 T cells, (**O**) Tcm, (**P**) B cells, (**Q**) T helper cells, (**R**) NK cells, (**S**) pDC, (**T**) Tem. A positive value of the correlation coefficient (r) indicates a positive relationship between the two variables, and on the contrary, a negative value indicates a negative relationship. Abbreviations: PD-L1: programmed death-ligand 1; ESCC: Esophageal Squamous Cell Carcinoma; aDC: Dendritic cells; DC: Dendritic cells; iDC: immature Dendritic cells; Tfh: T follicular helper; Tcm: T central memory; NK: Natural Killer cells; pDC: Plasmacytoid DC; Tem: T effector memory.
