# Supplementary material for: Prognostic significance of epidermal growth factor receptor and programmed cell death-ligand 1 co-expression in esophageal squamous cell carcinoma
Source: Aging (Albany NY). 2023 Feb 20;15(4):1107–29. doi: 10.18632/aging.204535 (PMC10008495; doi:10.18632/aging.204535)
Supplement: Supplementary Document 1 [file aging-15-204535-s002.pdf]

Supplementary Document 1. The differentially expressed EGFR in ESCC.

The differentially expressed EGFR in ESCC :

```
library(limma)
library(ggplot2)
library(ggpubr)

gene="EGFR"
expFile="symbol.txt"
setwd("C:\\bio\\Gene\\08.diff")
rt=read.table(expFile, header=T, sep="\t", check.names=F)
rt=as.matrix(rt)
rownames(rt)=rt[,1]
exp=rt[,2:ncol(rt)]
dimnames=list(rownames(exp),colnames(exp))
data=matrix(as.numeric(as.matrix(exp)),                nrow=nrow(exp),
dimnames=dimnames)
data=avereps(data)
data=t(data[gene,,drop=F])
group=sapply(strsplit(rownames(data),"\\-"), "[", 4)
group=sapply(strsplit(group,""), "[", 1)
group=gsub("2", "1", group)
conNum=length(group[group==1])
treatNum=length(group[group==0])
Type=c(rep(1,conNum), rep(2,treatNum))

exp=cbind(data, Type)
exp=as.data.frame(exp)
colnames(exp)=c("gene", "Type")
exp$Type=ifelse(exp$Type==1, "Normal", "Tumor")
exp$gene=log2(exp$gene+1)

outTab=exp
colnames(outTab)=c(gene, "Type")
outTab=cbind(ID=row.names(outTab), outTab)
write.table(outTab, file="geneExp.txt", sep="\t", quote=F, row.names=F)

group=levels(factor(exp$Type))
exp$Type=factor(exp$Type, levels=group)
comp=combn(group,2)
my_comparisons=list()
for(i in 1:ncol(comp)){my_comparisons[[i]]<-comp[,i]}
boxplot=ggboxplot(exp, x="Type", y="gene", color="Type",
                  xlab="",
                  ylab=paste0(gene, " expression"),
```

```
                  legend.title="Type",
                  palette = c("blue","red"),
                  add = "jitter")+
    stat_compare_means(comparisons=my_comparisons,symnum.args=list(cutpoints = c(0, 0.001, 0.01, 0.05, 1), symbols = c("***", "**", "*", "ns")),label = "p.signif")

pdf(file=paste0(gene,".diff.pdf"), width=5, height=4.5)
print(boxplot)
dev.off()
```
