# Supplementary material for: Prognostic significance of epidermal growth factor receptor and programmed cell death-ligand 1 co-expression in esophageal squamous cell carcinoma
Source: Aging (Albany NY). 2023 Feb 20;15(4):1107–29. doi: 10.18632/aging.204535 (PMC10008495; doi:10.18632/aging.204535)
Supplement: Supplementary Document 3 [file aging-15-204535-s004.pdf]

Supplementary Document 3. The detailed process drawing Lollipop plot.

The detailed process drawing Lollipop plots is as follows:

```
inputFile="cor.result.txt"
setwd("C:\\bio\\Gene\\27.Lollipop")

data = read.table(inputFile, header=T, sep="\t", check.names=F)

p.col = c('gold','pink','orange','LimeGreen','darkgreen')
fcolor = function(x,p.col){
  color =
ifelse(x>0.8,p.col[1],ifelse(x>0.6,p.col[2],ifelse(x>0.4,p.col[3],
                ifelse(x>0.2,p.col[4], p.col[5])
                )))
  return(color)
}

p.cex = seq(2.5, 5.5, length=5)
fcex = function(x){
  x=abs(x)
  cex =
ifelse(x<0.1,p.cex[1],ifelse(x<0.2,p.cex[2],ifelse(x<0.3,p.cex[3],
              ifelse(x<0.4,p.cex[4],p.cex[5]))))
  return(cex)
}
points.color = fcolor(x=data$pvalue,p.col=p.col)
data$points.color = points.color

points.cex = fcex(x=data$cor)
data$points.cex = points.cex
data=data[order(data$cor),]

xlim = ceiling(max(abs(data$cor))*10)/10
pdf(file="Lollipop.pdf", width=9, height=7)
layout(mat=matrix(c(1,1,1,1,1,0,2,0,3,0),nc=2),width=c(8,2.2),heights=
c(1,2,1,2,1))
par(bg="white",las=1,mar=c(5,18,2,4),cex.axis=1.5,cex.lab=2)
plot(1,type="n",xlim=c(-
xlim,xlim),ylim=c(0.5,nrow(data)+0.5),xlab="Correlation
Coefficient",ylab="",yaxt="n",yaxs="i",axes=F)
rect(par('usr')[1],par('usr')[3],par('usr')[2],par('usr')[4],col="#F5F
5F5",border="#F5F5F5")
grid(ny=nrow(data),col="white",lty=1,lwd=2)
segments(x0=data$cor,y0=1:nrow(data),x1=0,y1=1:nrow(data),lwd=4)
points(x=data$cor,y = 1:nrow(data),col =
```

```
data$points.color,pch=16,cex=data$points.cex)
text(par('usr')[1],1:nrow(data),data$Cell,adj=1,xpd=T,cex=1.5)
pvalue.text=ifelse(data$pvalue<0.001,'<0.001',sprintf("%.03f",data$pva
lue))
redcutoff_cor=0
redcutoff_pvalue=0.05
text(par('usr')[2],1:nrow(data),pvalue.text,adj=0,xpd=T,col=ifelse(abs
(data$cor)>redcutoff_cor                                              &
data$pvalue<redcutoff_pvalue,"red","black"),cex=1.5)
axis(1,tick=F)

par(mar=c(0,4,3,4))
plot(1,type="n",axes=F,xlab="",ylab="")
legend("left",legend=c(0.1,0.2,0.3,0.4,0.5),col="black",pt.cex=p.cex,p
ch=16,bty="n",cex=2,title="abs(cor)")

par(mar=c(0,6,4,6),cex.axis=1.5,cex.main=2)
barplot(rep(1,5),horiz=T,space=0,border=NA,col=p.col,xaxt="n",yaxt="n"
,xlab="",ylab="",main="pvalue")
axis(4,at=0:5,c(1,0.8,0.6,0.4,0.2,0),tick=F)
dev.off()
```
