# Supplementary material for: Prognostic significance of epidermal growth factor receptor and programmed cell death-ligand 1 co-expression in esophageal squamous cell carcinoma
Source: Aging (Albany NY). 2023 Feb 20;15(4):1107–29. doi: 10.18632/aging.204535 (PMC10008495; doi:10.18632/aging.204535)
Supplement: Supplementary Document 4 [file aging-15-204535-s005.pdf]

Supplementary Document 4. The detailed correlation analysis between EGFR expression and individual immune cell infiltration levels by the Spearman method.

The detailed correlation analysis between EGFR expression and individual immune cell infiltration levels by the Spearman method as follows:

```
library(limma)
library(ggplot2)
library(ggpubr)
library(ggExtra)

gene="EGFR"
pFilter=0.05
expFile="symbol.txt"
setwd("C:\\bio\\Gene\\17.cor")

rt=read.table(expFile, header=T, sep="\t", check.names=F)
rt=as.matrix(rt)
rownames(rt)=rt[,1]
exp=rt[,2:ncol(rt)]
dimnames=list(rownames(exp), colnames(exp))
data=matrix(as.numeric(as.matrix(exp)),                nrow=nrow(exp),
dimnames=dimnames)
data=avereps(data)
data=data[rowMeans(data)>1,]

group=sapply(strsplit(colnames(data),"\\-"), "[", 4)
group=sapply(strsplit(group,""), "[", 1)
group=gsub("2", "1", group)
data=data[,group==0]
data=log2(data+1)

x=as.numeric(data[gene,])
outTab=data.frame()
for(j in rownames(data)){
    if(gene==j){next}
    y=as.numeric(data[j,])
    corT=cor.test(x, y, method = 'Spearman')
    cor=corT$estimate
    pvalue=corT$p.value
    outTab=rbind(outTab, cbind(Query=gene, Gene=j, cor, pvalue))
    if((abs(cor)>corFilter) & (pvalue<pFilter)){
        df1=as.data.frame(cbind(x,y))
        p1=ggplot(df1, aes(x, y)) +
            xlab(paste0(gene,   "   expression"))+   ylab(paste0(j,   "
expression"))+
            geom_point()+   geom_smooth(method="lm",   formula=y~x)   +
```

```
theme_bw()+
            stat_cor(method = 'pearson', aes(x =x, y =y))
        pdf(file=paste0("cor.", j, ".pdf"), width=5, height=4.6)
        print(p1)
        dev.off()
    }
}

write.table(file="corResult.txt", outTab, sep="\t", quote=F, row.names=F)
outTab=outTab[abs(as.numeric(outTab$cor))>corFilter & as.numeric(outTab$pvalue)<pFilter,]
write.table(file="corSig.txt", outTab, sep="\t", quote=F, row.names=F)
```
