# Supplementary material for: Prognostic significance of epidermal growth factor receptor and programmed cell death-ligand 1 co-expression in esophageal squamous cell carcinoma
Source: Aging (Albany NY). 2023 Feb 20;15(4):1107–29. doi: 10.18632/aging.204535 (PMC10008495; doi:10.18632/aging.204535)
Supplement: Supplementary Document 6 [file aging-15-204535-s007.pdf]

Supplementary Document 6. The detailed calculation process of EGFR and PD-L1 immune cell infiltration in ESCC.

```
# library(tidyverse)
library(GSVA)
library(clusterProfiler)
library(org.Hs.eg.db)
library(data.table)
library(rtracklayer)

### ssGSEA ######
## table S1 - https://doi.org/10.1016/j.immuni.2013.10.003
## pdf -> table -> read
immunity <- read.csv("~/immunity-cell-gene.csv", header = T)
#   CellType AffymetrixID Symbol Gene.Symbol ENTREZ_GENE_ID
# 1      aDC    205569_at  LAMP3       LAMP3          27074
# 2      aDC    207533_at   CCL1        CCL1           6346
# 3      aDC    210029_at   INDO        IDO1           3620
# 4      aDC    218400_at   OAS3        OAS3           4940
# 5      aDC    219424_at   EBI3        EBI3          10148
# 6  B cells    204836_at   GLDC        GLDC           2731

idx <- !immunity$CellType %in% c("Blood vessels", "Normal mucosa", "
cancer cells", "Lymph vessels")
immunity <- immunity[idx,]
immunity <- immunity %>%
  split(., .$CellType) %>%
  lapply(., function(x)(x$ENTREZ_GENE_ID))
immunity <- lapply(immunity, unique)

## Ensembl download
anno <- import('~/Homo_sapiens.GRCh38.101.gtf')
anno <- as.data.frame(anno)
anno <- anno[!duplicated(anno$gene_id),]

anno <- merge(anno, gene_symbol, by = "gene_name")
anno <- rbind(anno, data.frame(gene_name = c("KIAA1324", "IGHA1"),
                               gene_id      =      c("ENSG00000116299",
"ENSG00000211895"),
                               ENTREZID = c("57535", "3492")))
anno <- anno[!duplicated(anno$gene_id),] ### 37417
anno <- anno[, c("gene_id", "ENTREZID")]


data <- fread("~/tpm.txt") %>%
  rename("gene_id" = "V1") %>%
  left_join(., anno, by = "gene_id") %>%
```

```
  filter(!is.na(ENTREZID)) %>%
  select(-gene_id) %>%
  column_to_rownames("ENTREZID")
data <- log2(data + 1)

immu_cell <-  as.data.frame(gsva(as.matrix(data), immunity, method =
"ssgsea"))



### bbt plot
data <- read.table("~/file.txt", header = T)
#        group       aDC     B cells CD8 T cells Cytotoxic cells
# 1 1.13092315 0.4709550  0.26202395   0.5944611       0.5130117
# 3 0.55644003 0.1800251 -0.07081909   0.5197230       0.2135559
# 4 0.44696904 0.3350859  0.05579749   0.5908900       0.3135561
# 5 0.05474605 0.1191767  0.02578815   0.5541712       0.2068595
# 7 0.61364297 0.1563856  0.09869185   0.5518254       0.2321028
# 8 0.41079217 0.4588979  0.50105493   0.5996277       0.4866508

data1 <- NULL
for(i in 2:25){
  cor <- cor.test(data[,i], data[,1], method = "pearson")
  data1 <- rbind(data1,
                 data.frame("group" = "a",
                            "cell" = colnames(data)[i],
                            "cor" = cor$estimate,
                            "p" = cor$p.value))
}
data1 <- data1[order(data1$cor),]
data1$cell <- factor(data1$cell, levels = data1$cell)

ggplot(data1, aes(x = cell, y = cor)) +
  geom_segment(aes(xend=cell,yend=0)) +
  geom_hline(yintercept = 0) +
  geom_point(aes(col=p, fill = p, size=abs(cor))) +
  coord_flip()
```
